# miR-143-3p Inhibits the Differentiation of Osteoclast Induced by Synovial Fibroblast and Monocyte Coculture in Adjuvant-Induced Arthritic Rats

**DOI:** 10.1155/2021/5565973

**Published:** 2021-08-26

**Authors:** Baoping Jiang, Chengchen Yuan, Jing Han, Meiyu Shen, Xueping Zhou, Lingling Zhou

**Affiliations:** ^1^School of Pharmacy, Nanjing University of Chinese Medicine, Nanjing, China; ^2^The First Clinical Medical College, Nanjing University of Chinese Medicine, Nanjing, China

## Abstract

Osteoclast, which mediates overactive bone resorption, is one of the key factors for bone destruction in rheumatoid arthritis (RA). Existing studies have shown that abnormal miR-143-3p expression was observed in both RA patients and arthritis animals, which can participate in osteoclast differentiation, and mitogen-activated protein kinase (MAPK) signaling pathway was closely related to osteoclast differentiation. The primary objective of the current study was to determine the role of miR-143-3p in the progression of osteoclast differentiation and its relationship with MAPK signaling pathways. The results showed that miR-143-3p inhibited osteoclast differentiation and decreased the levels of M-CSF and RANKL during osteoclast differentiation. miR-143-3p inhibited the expression of MAPK signaling proteins, which is ERK1/2 in the early stage and JNK in the later stage of osteoclast differentiation. It was also observed that MAPK inhibitors upregulated miR-143-3p expression in osteoclast differentiation. Taken together, our results suggested that miR-143-3p could inhibit the differentiation of osteoclast, which was related to inhibiting MAPK signaling pathways. This may provide a novel strategy for curing RA.

## 1. Introduction

RA is a chronic systemic autoimmune disease. It is well known that bone destruction can occur in the early stages of RA. Osteoclast, which can mediate overactive bone resorption, is considered to be one of the key cells that cause bone destruction in RA [1]. Many researches have focused on the mechanisms of osteoclast differentiation to develop the new strategies of RA treatment.

Studies have shown that the bone destruction is closely related to synovitis in RA [2]. Synovial cells can secrete various kinds of cytokines such as macrophage colony-stimulating factor (M-CSF) and express osteoclast differentiation factor (ODF)/receptor activator of NF-kappa B ligand (RANKL) [3, 4], which can initiate a variety of intracellular signaling transduction pathways that regulate osteoclast-specific gene expression and transcription including MAPK, thereafter to induce osteoclast differentiation and lead to bone destruction [5].

More researches suggested that miRNA played a key role in the complex pathogenesis of RA [6]. They could regulate the pathological processes of synovitis and bone destruction in RA by affecting cytokines and signaling molecules [7, 8]. Our previous studies found that there was abnormal expression of miR-143-3p in the plasma of RA patients and arthritis animals, and low expression of miR-143-3p was also found in the cocultured osteoclast-like cells [9]. It has been reported that miR-143-3p can affect the secretion of osteoclast differentiation-related cytokines [10]. Meanwhile, bioinformatics analysis of miR-143-3p showed that mitogen-activated protein kinase (MAPK) signaling pathways were closely related to osteoclast differentiation [9]. It has been reported that miR-143 inhibited the differentiation of RAW264.7 monocytes to osteoclasts [11], but there is no research on the process of osteoclasts induced by synovial fibroblasts and monocytes in RA. In the previous study, we established a method of inducing osteoclasts by coculture of synovial fibroblasts and peripheral blood monocytes from adjuvant-induced arthritis (AIA) rats, which can better reflect the inner link between synovitis and bone destruction of RA.

To determine the role of miR-143-3p in the progression of osteoclast differentiation and the specific mechanism, the synovial fibroblasts and peripheral blood monocytes from AIA rats were cocultured to induce osteoclasts. miR-143-3p mimics/inhibitors were separate used, and MAPK inhibitors were used in this study. Our data indicated that miR-143-3p could inhibit the differentiation of osteoclast, and its expression was negatively correlated with MAPK protein expressions. This study clarified that miR-143-3p could inhibit the osteoclast differentiation, which was related to inhibiting MAPK signaling pathways. This study is helpful for the study on the therapeutic drugs of RA and may provide a novel strategy for the clinic treatment of RA.

## 2. Methods and Materials

### 2.1. RA Patient Collection

All experiment protocols were approved by the Jiangsu Province Hospital of Chinese Medicine. Written consent was obtained from participants. A total of five healthy controls (HCs) (2 males and 3 females; the average age was 55.6 years), and five RA severe patients (2 males and 3 females; the average age was 56.2 years; according to the modified international DAS28 score [12], we choose RA patients with DAS28‐ESR > 5.1 for this study) were included in the present study (ethics approval number: 2016NL-KS14).

### 2.2. Animals

Male SD rats weighing 180-220 g were purchased from Zhejiang Experimental Animal Center (Zhejiang, China). All animals were maintained under specific pathogen-free conditions at the Nanjing University of Chinese Medicine Experimental Animal Center. All experiment processes were in strict accordance with the regulations on the use and management of experimental animals of Nanjing University of Chinese Medicine.

### 2.3. AIA Model Preparation

Four male SD rats weighing 180-220 g were purchased from Zhejiang Experimental Animal Center (Zhejiang, China). All animals were maintained under specific pathogen-free conditions at the Nanjing University of Chinese Medicine Experimental Animal Center. The experimental animals were acclimated for one week before the beginning of the study with free access to rodent diet and water. All experiment processes were in strict accordance with the regulations on the use and management of experimental animals of Nanjing University of Chinese Medicine. Two male SD rats were used to prepare the AIA rat model as previously described [13]. Complete Freund's adjuvant (CFA) (including BCG (Beijing Institute of Biological Products Co. Ltd., Beijing, China) 7.5 mg/mL) was prepared, and the model of AIA was established by intradermal injection of CFA (0.05 mL) into the right hind foot of SD rats. The other two male SD rats were used as controls.

### 2.4. Establishment of Coculture System of Peripheral Blood Monocytes and Synovial Fibroblasts from AIA Rats to Induce Osteoclast-Like Cells

Osteoclast-like cells were induced according to the method which we have founded in the previous study [13]. The fresh anticoagulant blood of AIA rats was isolated with lymphocyte separation solution, transferred into the cell bottle, and cultured for about 12 hours. The upper lymphocytes were discarded, and the lower adherent cells were mononuclear cells. Then, AIA rats were sacrificed, the synovial tissue of the joint was separated, 15% NBS-DMEM was added, and it was then cut into pieces (1-2 mm^3^) and carefully moved into the culture flask, 5% CO_2_ was adhered to the wall at 37°C for 4 hours, and 15% NBS-DMEM 5% CO_2_ was added to continue the culture at 37°C. The culture medium was changed every three days and passed on after the culture bottle was full.

The monocytes were isolated from the peripheral blood of AIA rats, and the synovial fibroblasts of AIA rats were cultured and transmitted to 3-5 generations. In addition, the monocytes were isolated from fresh normal male rats' anticoagulant blood as control. 2 mL of monocytes (1 × 10^6^ mL^−1^) was added to the 6-well plates. Synovial fibroblasts in the logarithmic growth phase were prepared into 1 × 10^5^ mL^−1^ cell suspension, which were added to the hanging Transwell chamber in the 6-well plates. The cells were cocultured in a 5% CO_2_ incubator at 37°C to induce osteoclast-like cells. The solution was changed every other day. After 3 weeks, the cells were identified by tartrate-resistant acid phosphatase (TRAP) staining.

### 2.5. Detection of miR-143-3p Expression Using qPCR

Total RNA of samples was isolated using the TRIzol reagent (Austin, TX, USA) and performed with miRNA 1st Strand cDNA Synthesis Kit (by stem-loop) or reverse transcription kit HiScript Q RT SuperMix (Vazyme, Nanjing, China) to generate cDNA. Then, qPCR reactions were performed using SYBR Green qPCR Master Mix (Vazyme, Nanjing, China) according to the manufacturer's instructions.

The following primers (Sangon Biotech Co., Ltd., Shanghai, China) were used for amplification: U6—forward 5′-GCTTCGGCAGCACATATACTAAAAT-3′ and reverse 5′-CGCTTCACGAATTTGCGTGTCAT-3′; hsa-miR-143-3p sequence-specific primers—forward 5′-GGGATGAGATGAAGCACT-3′ and reverse 5′-GTGCGTGTCGTGGAGTCG-3′; rno-miR-143-3p sequence-specific primers—forward 5′-GGGTTGAGATGAAGCACTGT-3′ and reverse 5′-CAGTGCGTGTCGTGGAG-3′; and mmu-miR-143-3p sequence-specific primers—forward 5′-CGCGTGAGATGAAGCACTG-3′ and reverse 5′-AGTGCAGGGTCCGAGGTATT-3′.

The qPCR reactions were performed on an ABI 7500 thermocycler (Applied Biosystems Life Technologies, Foster City, CA, USA). Data were analyzed using the comparative Ct method (2^-∆∆Ct^).

### 2.6. Detection of Cytokine Level Using Enzyme-Linked Immunosorbent Assay (ELISA)

The culture supernatants of coculture cells were collected to determine the level of TRAP, M-CSF, and RANKL by ELISA kits (Shanghai Yuanye Bio-Technology Co., Ltd., Shanghai, China).

### 2.7. Cell Transfection

Transfection reagents Lipofectamine RNAiMAX (Invitrogen, Carlsbad, CA, USA), miR-143-3p mimics NC (Shanghai GenePharma Co., Ltd., Shanghai, China), miR-143-3p mimics (Shanghai GenePharma Co., Ltd., Shanghai, China), miR-143-3p inhibitors NC (Shanghai GenePharma Co., Ltd., Shanghai, China), and miR-143-3p inhibitors (Shanghai GenePharma Co., Ltd., Shanghai, China) were diluted to a concentration of 10 *μ*M according to the instructions.

Coculture cells were transfected with Lipofectamine™ RNAiMAX transfection reagent (Invitrogen, Carlsbad, CA, USA). Lipofectamine™ RNAiMAX (6 *μ*L), miR-143-3p mimics (2 *μ*L), miR-143-3p mimics NC (2 *μ*L), miR-143-3p inhibitors (2 *μ*L), and miR-143-3p inhibitors NC (2 *μ*L) were diluted with 150 *μ*L Opti-MEM and mixed well. The diluted target gene (152 uL) and diluted Lipofectamine™ RNAiMAX (156 *μ*L) were mixed and placed at room temperature for 5 minutes. In the early stage of osteoclast differentiation (coculture for 7 days) and later stage (coculture for 14 days), the prepared transfection reagent (308 uL) was added and then shaken and mixed gently and cultured in a CO_2_ incubator at 37°C (cells continued to be cultured for 48 hours in the early differentiation stage and 24 hours in the later stage of differentiation), and then the transfection and cell status were observed. The supernatant of the cells was collected, and cellular RNA and proteins were extracted for detection.

### 2.8. Detection of the Expressions of MAPK Proteins Using Western Blot [14]

The osteoclasts induced by coculture of synovial fibroblasts and monocytes from AIA rats were collected and washed twice with PBS. The expression of p38 mitogen-activated protein kinase (p38MAPK), extracellular signal-regulated kinase (ERK1/2), c-Jun NH2-terminal kinase (JNK), phosphorylation p38 mitogen-activated protein kinase (p-p38MAPK), p-ERK1/2, and p-JNK (Danvers, MA, USA) in osteoclasts was detected by the western blot method.

### 2.9. Statistical Analysis

Quantitative data were presented as the mean ± standard deviation (SD). Unpaired Student's *t*-tests, the Kruskal and Wallis test, and one-way ANOVA with Tukey's studentized range test were used to analyze data. Date analysis was performed using GraphPad Prism 8.0. *p* values < 0.05 were considered statistically significant.

## 3. Results

### 3.1. Low Expression of miR-143-3p Was Found in Both RA Patients and Cocultured Osteoclast-Like Cells

**Figure 1:**
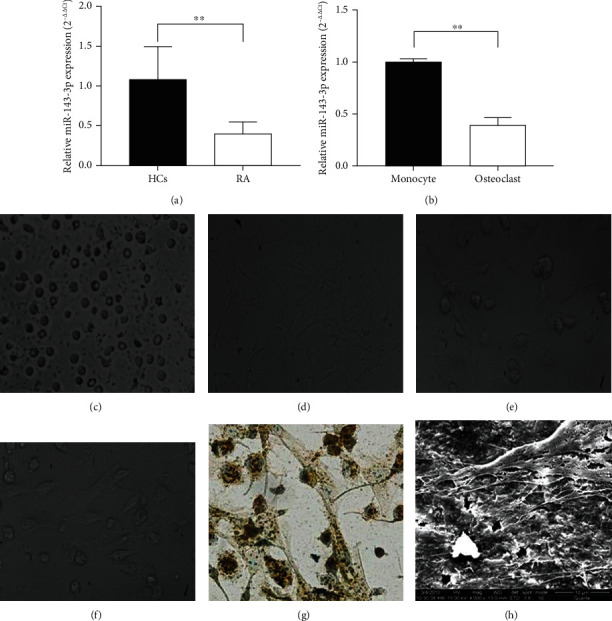
Osteoclast differentiation, morphological characteristics, and miRNA-143-3p expression in RA and osteoclast-like cells. (a) miR-143-3p expression in RA patients and healthy controls determined by qPCR analysis (data were expressed as the means ± SD of five independent experiments). (b) miR-143-3p expression in normal monocytes and osteoclasts detected by qPCR analysis (data were expressed as the means ± SD of three independent experiments). The monocytes (c) were cocultured with synovial fibroblasts (d) of AIA rats, and the monocytes began to differentiate after 5-7 days of coculturing (multinucleated cells appeared with flaky or filamentous pseudopodia) (e) (200x magnification). They differentiated into osteoclast-like cells after 12-14 days of coculturing (f) (200x magnification). (g) Osteoclast-like cells were identified as osteoclasts by TRAP staining (200x magnification). (h) A scanning electron microscope showed that osteoclast-like cells had bone resorption function (4000x magnification). ^∗∗^*p* < 0.01 compared with the control group.

In the present study, the plasma expression of miR-143-3p in RA patients and HCs was analyzed by qPCR, and the results showed that the expression of miR-143-3p in peripheral blood of RA patients was significantly lower than that of HCs (Figure 1(a)).

The monocytes were cocultured with synovial fibroblasts, the cells began to differentiate after 7 days of coculturing, and they differentiated into osteoclast-like cells after 14 days (Figures 1(c)–1(f)). After being cocultured with fibroblasts for 14 days, monocytes were identified to differentiate into osteoclasts by TRAP staining (Figure 1(g)). A scanning electron microscope showed that osteoclasts attached and grew on the bone slices, protruding flaky, or filamentous pseudopodia, and bone resorption lacunae of different sizes appeared on the bone slices, indicating that osteoclasts cocultured with monocytes and synovial fibroblasts had obvious bone resorption function (Figure 1(h)).

Subsequently, the expression of miR-143-3p in osteoclasts and normal monocytes was detected by qPCR. The results showed that the expression of miR-143-3p in osteoclasts was significantly lower than that in the normal monocytes (Figure 1(b)), which was consistent with the low expression of miR-143-3p in RA patients.

All the results indicated that low expression of miR-143-3p is a common characteristic in the arthritis of patients and animals.

### 3.2. miR-143-3p Expression Was Negatively Correlated with Osteoclast Differentiation

**Figure 2:**
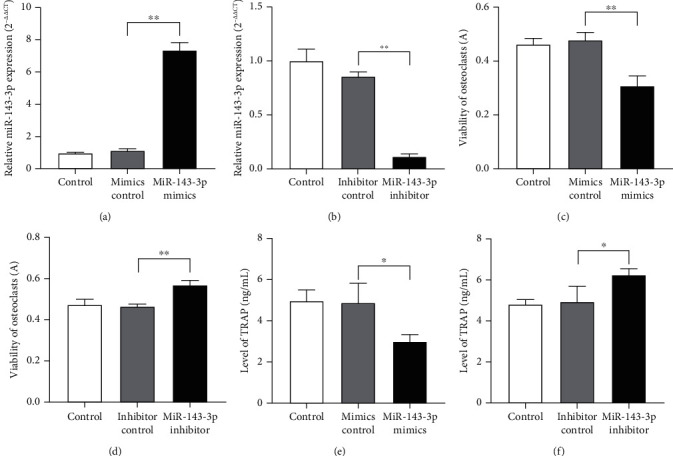
miR-143-3p expression was negatively correlated with osteoclast differentiation. Expression of miR-143-3p in osteoclasts transfected with miR-143-3p mimics or inhibitors (a, b) determined by qPCR analysis. Viability of osteoclasts transfected with miR-143-3p mimics or inhibitors (c, d) was measured by ELISA. TRAP level of osteoclasts transfected with miR-143-3p mimics or inhibitors (e, f) was detected by ELISA. Data were expressed as the means ± SD of three independent experiments. ^∗^*p* < 0.05 and ^∗∗^*p* < 0.01 compared with the negative control group.

Osteoclast-like cells that induced after coculture of monocytes and synovial fibroblasts for 14 days were transfected with miR-143-3p mimics, inhibitors, and negative control RNA, respectively. The results of miR-143-3p expression verified the success of transfection (Figures 2(a) and 2(b)).

Meanwhile, osteoclast viability was measured by the 3-[4,5-dimethylthiazol-2-yl]-2,5-diphenyl tetrazolium bromide (MTT) method, and the TRAP level of osteoclasts was detected by ELISA. The results showed that the viability and TRAP level of osteoclasts decreased after transfection with miR-143-3p mimics (Figures 2(c) and 2(e)), and the results were on the contrary after transfection with inhibitors (Figures 2(d) and 2(f)). This indicated that miR-143-3p was negatively correlated with osteoclast differentiation.

### 3.3. miR-143-3p Regulated the Differentiation-Related Cytokine Secretion and the Activation of MAPK Proteins in the Process of Osteoclast Differentiation

**Figure 3:**
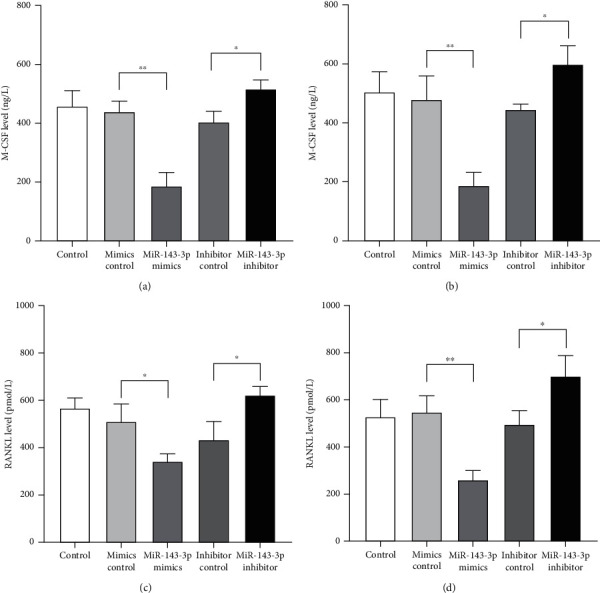
miR-143-3p expression was negatively correlated with differentiation-related cytokines secretion. Levels of M-CSF and RANKL in culture supernatant of cocultured cells which transfected with miR-143-3p mimics or inhibitors in the early (coculture for 7 days) or later stage (coculture for 14 days) of osteoclast differentiation were detected by ELISA. M-CSF levels in culture supernatant of cocultured cells in the early (a) or later (b) stage of osteoclast differentiation. RANKL levels in culture supernatant of cocultured cells in the early (c) or later (d) stage of osteoclast differentiation. Data were expressed as the means ± SD of three independent experiments. ^∗^*p* < 0.05 and ^∗∗^*p* < 0.01 compared with the negative control group.

The levels of M-CSF and RANKL, which were key factors to the osteoclast differentiation, were detected in the supernatants of cocultured cells after transfection in the early (coculture for 7 days) or later (coculture for 14 days) differentiation stage. As Figures 3(a)–3(d) have shown, in the early and later stages of osteoclast differentiation, the levels of M-CSF and RANKL significantly decreased when being transfected with miR-143-3p mimics, but increased in those transfected with miR-143-3p inhibitors.

Previous bioinformatics analysis indicated that MAPK might be one of the target pathways of miR-143-3p [9]. Thus, we investigated the regulating effect of miR-143-3p on MAPK activation in the process of monocytes differentiating and fusing into osteoclasts.

**Figure 4:**
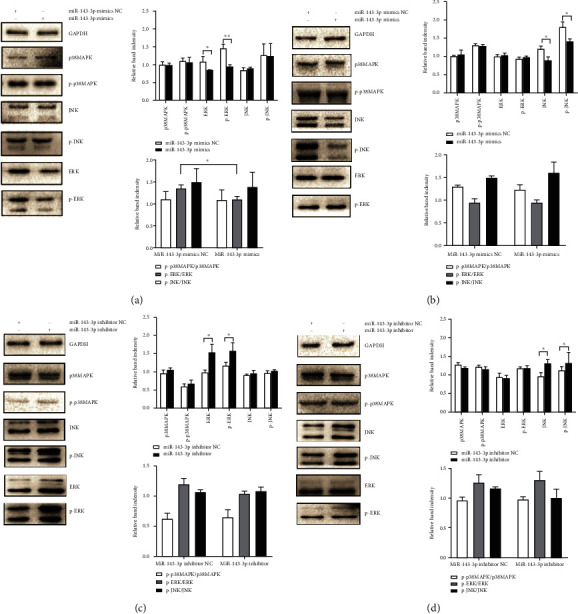
miR-143-3p regulated the activation of MAPK proteins in the process of osteoclast differentiation. The expression of JNK, ERK1/2, p38MAPK, p-JNK, p-ERK1/2, and p-p38MAPK in osteoclasts was detected by western blot after miR-143-3p mimics were transfected in the early (coculture for 7 days) (a) or later (coculture for 14 days) (b) stage of osteoclast differentiation. The expression of JNK, ERK1/2, p38MAPK, p-JNK, p-ERK1/2, and p-p38MAPK in osteoclasts was detected after miR-143-3p inhibitors were transfected in the early (c) or later (d) stage of osteoclast differentiation. Data were expressed as the means ± SD of three independent experiments. ^∗^*p* < 0.05 and ^∗∗^*p* < 0.01 compared with the negative control group.

The expressions of MAPK signaling pathway-related proteins and phosphorylation proteins in osteoclasts were detected after miR-143-3p mimics and inhibitor transfection. The results showed that in the early stage of differentiation, miR-143-3p mimics inhibited the expression of ERK1/2 and p-ERK1/2 (Figures 4(a) and 4(c)), while miR-143-3p inhibitors promoted the expressions of ERK1/2 and p-ERK1/2. In the later stage, miR-143-3p mimics inhibited the expression of JNK and p-JNK, while miR-143-3p inhibitors promoted the expressions (Figures 4(b) and 4(d)).

These results suggested that miR-143-3p was negatively correlated with the level of differentiation-related M-CSF and RANKL in different stages of osteoclast differentiation. In addition, miR-143-3p was negatively correlated with the activation of MAPK proteins in osteoclast differentiation by acting on ERK1/2 protein in the early stage and JNK protein in the later stage.

### 3.4. MAPK Inhibitors Inhibited the Osteoclast Differentiation and Upregulated miR-143-3p Expression

It has been reported that MAPK signaling pathways could affect the miR-143-3p expression [15], so we further investigated if MAPK signaling pathways could also influence miR-143-3p expression in the process of monocytes differentiating and fusing into osteoclasts using the MAPK inhibitors, including ERK1/2 inhibitor (U0126), JNK inhibitor (SP600125), and p38MAPK inhibitor (SB203580) (Beyotime, Shanghai, China).

**Figure 5:**
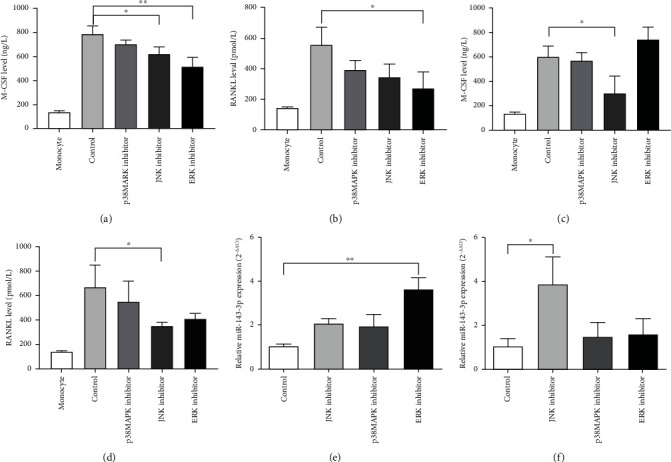
MAPK inhibitors reduced the level of cytokines in culture supernatants of coculture cells and upregulated the expression of miR-143-3p in osteoclasts. Levels of M-CSF and RANKL in culture supernatants of coculture cells in the early (coculture for 7 days) (a, b) or later (coculture for 14 days) (c, d) stage of osteoclast differentiation with MAPK inhibitors detected by ELISA. miR-143-3p expression of osteoclasts in the early (e) or later (f) stage of differentiation with MAPK inhibitors by qPCR analysis. Data were expressed as the means ± SD of three independent experiments. ^∗^*p* < 0.05 and ^∗∗^*p* < 0.01 compared with the control group.

The results showed that MAPK inhibitors reduced the levels of M-CSF and RANKL in culture supernatant of cocultured cells, and the effect of ERK1/2 inhibitor was significantly in the early stage (coculture for 7 days) (Figures 5(a) and 5(b)), while JNK inhibitor had the significant effect in the later stage (coculture for 14 days) (Figures 5(c) and 5(d)).

Meanwhile, miR-143-3p expression in osteoclasts was upmodulated with MAPK inhibitors treatment. ERK1/2 inhibitor had a significant effect in the early stage (Figure 5(e)), while the effect of JNK inhibitor was significantly in the later stage (Figure 5(f)). This indicated that the MAPK pathway also had a modulatory effect on miR-143-3p expression, and they might have interactive regulations that participated in the process of monocytes differentiating and fusing into osteoclasts.

## 4. Discussion

RA is a chronic autoimmune disease characterized by erosion of the joints. In the process of bone destruction in RA, the excessive proliferation of osteoclasts breaks the balance between osteoblasts and osteoclasts, and bone destruction accordingly occurs. Many factors, such as cytokines and signaling proteins, affect the differentiation of osteoclasts. They have complex interactions with each other, among which miRNAs may play a key role. In this study, we found the abnormal expression of miR-143-3p in RA patients and monocytes during differentiation and fusion into multinucleated osteoclasts. Moreover, in the process of osteoclast differentiation, miR-143-3p was negatively correlated with MAPK signaling proteins, which jointly affected cytokine secretion and thus interfered with osteoclast differentiation.

MicroRNA is a class of small noncoding RNA, which plays a key role in cancer, inflammation, and nervous system diseases by regulating protein expression [16–18]. We used miRNA microarray to screen the expression of miRNAs in plasma of RA patients, AIA rats, and CIA mice and found a variety of abnormal expression of miRNA, among which the miR-143-3p showed low expression in RA patients and various animal models [9, 19]. It has also been reported that the expression of miR-143 was downregulated in RA fibroblast synovium cells stimulated by type II collagen [20]. There was a report that miR-143 could inhibit mouse RAW264.7 monocytes differentiating and fusing into multinucleated osteoclasts [11]. Thus, we further investigated the expression of miR-143-3p in osteoclasts, and to clarify the role of miR-143-3p in the process of monocytes differentiating and fusing into osteoclasts, miR-143-3p mimics and inhibitors were used simultaneously in the experiment.

RA is associated with excessive proliferation and differentiation of osteoclasts. Monocytes and synovial fibroblasts play an important role in the process of osteoclast differentiation and proliferation. Existing research methods on osteoclasts mainly use inducers to induce monocytes to differentiate into osteoclasts, but this method cannot reflect the process of osteoclast proliferation and differentiation in RA disease. In our previous study, a coculture system had been successfully established to induce osteoclasts, in which synovial fibroblasts of AIA rats were served as stromal cells and peripheral blood monocytes of AIA rats were served as osteoclast precursor cells [5]. In the process of coculturing, the cytokines secreted by synovial fibroblasts could stimulate monocytes to differentiate into osteoclasts without adding exogenous stimulating factors. Monocytes began to differentiate after 7 days of coculture and could differentiate into osteoclast-like cells in 2-3 weeks. This method of osteoclasts induction could better reflect the pathological process of arthritis.

In the present study, the coculture system was used, and the low miR-143-3p expression was observed in osteoclast-like cells, which was consistent with that of RA patients and arthritis animals. However, there are also some contrary results of miR-143-3p expression [15] in some synovial fluid or the cell line. We noted that the number of clinical cases in the literature is not big. In the case of limited sample size of cases, the researchers may observe different results, and the scholars thought they may be related with different stages of RA and different tissues and cells [21]. Low expression of miR-143-3p had been verified in our experiment. Our results also showed that the viability and TRAP level of osteoclasts decreased after miR-143-3p mimic transfection, while they increased after miR-143-3p inhibitor transfection, which indicated that miR-143-3p was negatively correlated with osteoclast differentiation. All the results indicated that miR-143-3p could inhibit the differentiation of osteoclast.

Then, we investigated the mechanism of miR-143-3p participating in the process of osteoclast differentiation. As we have known, the cytokine network, including M-CSF and RANKL, plays an important role in the process of osteoclast differentiation [22]. miR-143-3p mimics and inhibitor were transfected into the coculture system, and our results indicated that miR-143-3p expression was negatively correlated with the levels of RANKL and M-CSF at different stages of osteoclast differentiation, suggesting that miR-143-3p could regulate the secretion of cytokines related to osteoclast differentiation during the whole process.

MAPKs (ERK1/2, p38MAPK, and JNK) were important regulatory signaling pathways which stimulate the differentiation, survival, and fusion of osteoclast precursors and activate mature osteoclasts [5]. Moreover, the MAPK pathway could participate in the occurrence and development of cartilage and bone destruction through the RANK-RANKL-osteoprotegerin (OPG) signaling pathway [23]. Further studies showed the relationship between miR-143-3p and cytokines or MAPK signaling pathways [9]. Therefore, the effects of miR-143-3p on MAPKs were investigated at the early and later stages of differentiation, respectively. Our results showed that miR-143-3p could negatively regulate the expression of MAPK proteins, but its regulatory proteins were different in the early and later stages of osteoclast differentiation. miR-143-3p mainly regulated the expression and phosphorylation of ERK1/2 in the early stage (coculture for 7 days), while it modulated the expression and phosphorylation of JNK in the later stage (coculture for 14 days) of osteoclast differentiation. In addition, we found that MAPK inhibitors could inhibit the levels of M-CSF and RANKL in the supernatant of coculture cells at both the early and later stages of differentiation. The effect of ERK inhibitor was more obvious in the early stage of differentiation, and the effect of JNK inhibitor was more obvious in the late stage of differentiation, which was consistent with the MAPK target of miR-143-3p in the early and late stages of differentiation. These results indicated that miR-143-3p may also inhibit osteoclast differentiation by regulating the MAPK pathway in the whole process of differentiation.

Interestingly, we also found that MAPK inhibitors upregulated the miR-143-3p expression. It has been reported that MAPK signaling pathways can regulate the miR-143 expression [24]. Considering all the results, we supposed that miR-143-3p was negatively correlated with the MAPK signaling pathways, and they interacted with each other to regulate the differentiation and fusion of monocytes into osteoclasts. It has been reported that MAPK inhibitors alone are not very effective in RA treatment [25]. Our results indicated that intervention of miR-143-3p and MAPK pathways in the process of differentiation and fusion of monocytes into osteoclasts might be the effective measure of curing RA.

In conclusion, our results demonstrated that miR-143-3p was negatively correlated with osteoclast differentiation. miR-143-3p interacts with the MAPK pathway inhibit the secretion of cytokines related to osteoclast differentiation and affect the process of differentiation and fusion of monocytes into osteoclasts. Moreover, at different stages of osteoclast differentiation, miR-143-3p negatively regulated the ERK and JNK MAPK signaling pathways. We will further design the *in vivo* experiment using arthritis animals to verify the effects of miR-143-3p on the symptoms of arthritis and bone destruction. The specific mechanism of the interaction between miR-143-3p and MAPK in regulating osteoclast differentiation will also be studied in the future. Our findings are helpful not only for the study on the pathological mechanism and therapeutic drugs of RA but also for the study of bone diseases related to osteoclast differentiation.

## Data Availability

The datasets generated during and/or analyzed during the current study are available from the corresponding author on reasonable request.
